# Draft genome of pin nematode *Paratylenchus projectus* recovered from rhizosphere of blueberry

**DOI:** 10.1186/s13071-025-06680-8

**Published:** 2025-02-26

**Authors:** Liang-Qin Liu, Wei-Qi Fu, Yuan-Yuan Ma, Zhi-Yin Liu, Chun-feng Ge, Yi-Ru Yang, Xue Qing, Qi-Long Zeng

**Affiliations:** 1 Jiangsu Key Laboratory for the Research and Utilization of Plant Resources, Institute of Botany, Jiangsu Province and Chinese Academy of Sciences (Nanjing Botanical Garden Mem. Sun Yat-Sen), Nanjing, 210014 China; 2 Jiangsu Engineering Research Center for the Germplasm Innovation and Utilization of Blueberry, Nanjing, 210014 China; 3 Department of Plant Pathology, Nanjing Agricultural University, Nanjing, 210095 China; 4 Ningxia Rural Science and Technology Development Center, Yinchuan, 750001 Ningxia China

**Keywords:** Glycoside hydrolase family 18, Glycoside hydrolase family 5, Tylenchulidae, Genome sequencing

## Abstract

**Background:**

The pin nematode, belonging to the genus *Paratylenchus*, parasitizes higher plants, often causing reduced or inhibited root tip development.

**Methods:**

Pin nematodes were isolated from the roots and rhizosphere of blueberry plants and subsequently identified as representatives of *Paratylenchus projectus* based on morphological characteristics and molecular barcoding. The *P. projectus* draft genome was sequenced using the Illumina platform.

**Results:**

Phylogenetic analysis based on 18S, 28S and ITS rRNA placed this species in highly supported clades alongside other *P. projectus* specimens. The draft genome of *P. projectus* was sequenced and assembled, representing the first genomic data for both the genus *Paratylenchus* and the family Tylenchulidae. The assembled genome, though fragmented, had a total length of 191.36 Mb and an estimated genome size of 64.9 Mb. Protein-coding genes were predicted using four different databases, with particular focus on carbohydrate-active enzymes from the GH5 and GH18 families. The recovered GH5 genes were distributed among three distinct clades: one forming a basal group relative to other nematodes, one as a sister clade to the fungivorous nematode *Aphelenchus avenae* and one nested within a fungal clade. The GH18 chitinase genes were grouped into two clades: one closely related to sedentary plant-parasitic nematodes of the genera *Heterodera* and *Globodera* and the other closely related to the fungivorous nematode *Ditylenchus*.

**Conclusions:**

The draft genome of *Paratylenchus projectus* was sequenced and assembled, representing the first genomic data for both the genus *Paratylenchus* and the family Tylenchulidae to our knowledge.

**Graphical Abstract:**

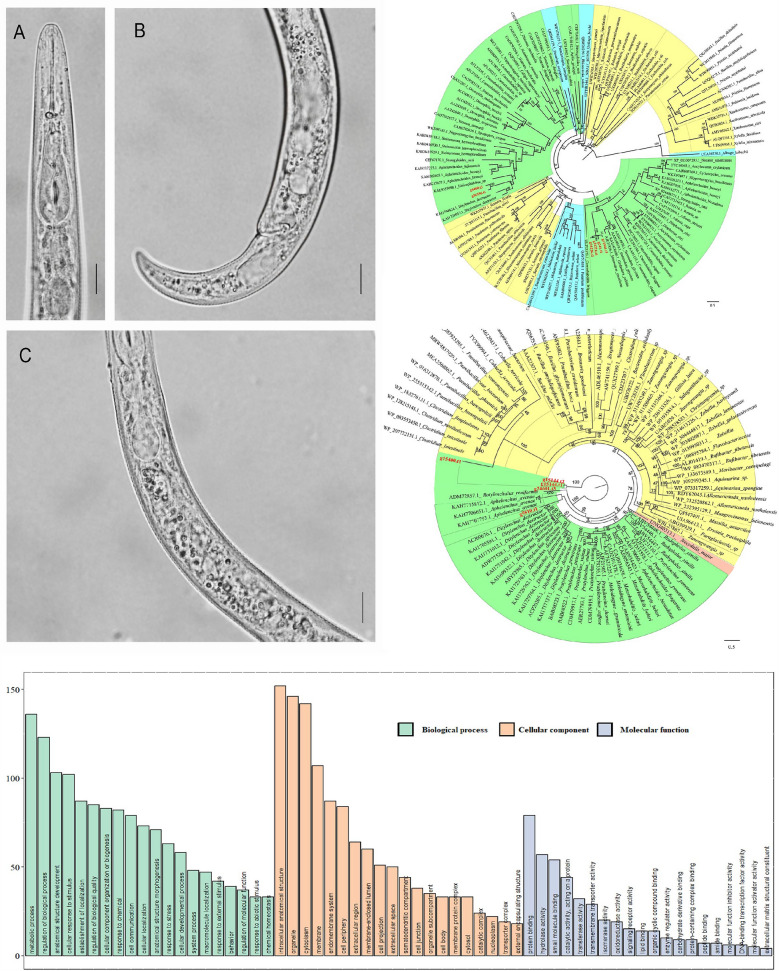

**Supplementary Information:**

The online version contains supplementary material available at 10.1186/s13071-025-06680-8.

## Background

The blueberry (*Vaccinium corymbosum* L.) is native to North America and is among the most economically significant fruit crops globally [1]. Previous studies have shown that several plant-parasitic nematodes (PPNs) cause damage to blueberry roots. For instance, blueberry stubby root nematodes (*Trichodorus* spp. and *Paratrichodorus* spp.) have been found in the rhizosphere soil of major growing regions across North America [2]. Forge et al. demonstrated that highbush (*V. corymbosum*) and lowbush (*Vaccinium angustifolium*) blueberries are good hosts for *Paratrichodorus renifer*, which leads to reduced yield, smaller canopy volume and lower top dry weight, indicating that *P. renifer* can significantly damage blueberry plants [3]. In both greenhouse and field conditions, the ring nematode *Mesocriconema ornatum* causes stunted growth, yellowing and yield loss in blueberry [4]. However, there have been no reports of pin nematodes affecting blueberries.

Pin nematodes, belonging to the genus *Paratylenchus*, parasitize higher plants, particularly in the rhizosphere of fruit trees [5]. These nematodes use long stylets to feed on epidermal cells or cortical tissue, which reduces the roots' absorption capacity and promotes root death [6]. The genus *Paratylenchus* represents a unique lineage for plant-parasitic nematode, as in molecular phylogeny it was placed within Criconematoidea, next to morphologically distinct Tylenchulidae and Criconematidae [7] but far from either more derived plant parasites like root-knot nematode or basal clades like stem nematode *Ditylenchus*. Therefore, this species represents an intermediate form from obligated plant parasites to frugivorous Tylenchomorpha, and its feeding-related genes are thus valuable in understanding the evolution plant parasitism.

In this study, pin nematodes were isolated from the roots and rhizosphere of blueberry plants and subsequently identified based on morphological characteristics and molecular barcoding. The draft genome was sequenced using the Illumina platform. The genome was assembled and annotated to investigate the gene composition, with special focus on carbohydrate-active enzymes. Our results provide the first genomic representation for the pin nematode and the family Tylenchulidae to our knowledge.

## Methods

### Sampling and species identification

Blueberry rhizosphere soil was collected from a blueberry field in Heilongjiang, China (GPS coordinates: 47°53′23.685ʺN, 128°54′56.713ʺE). Nematodes were extracted using a Baermann tray [8], collected and concentrated using a 500-mesh sieve (25-μm opening). After removing water, nematodes were placed in 20 μL distilled water on a slide. The slide was heated to fix the specimen, covered with a coverslip and sealed with nail polish. Photographs were made using an Olympus BX51 DIC Microscope (Olympus Optical, Tokyo, Japan) with an Olympus C5060Wz camera.

### DNA extraction, sequencing and phylogeny reconstruction

After species identification, genomic DNA extraction and amplification were performed with the REPLI-g Single Cell Kit (Qiagen, Germany) using five live individuals following the kit's user manual. The TruSeq DNA Sample Prep Kit (Illumina Inc., CA, USA) was used for library preparation according to the manufacturer’s instructions. Sequencings were performed using the Illumina NovaSeq PE150 platform.

rRNA was extracted and assembled from raw reads in genome sequencing. In brief, the rRNA in raw reads was identified by aligning it against a nematode rRNA reference using NextGenMap [9]. The aligned reads were extracted using SAMtools [10] and subsequently assembled in Megahit [11]. The resulting contigs were aligned with rRNA references in NCBI in Geneious 7.13 to locate the 18S rRNA, 28S rRNA and internal transcribed spacer (ITS) sequences. The newly obtained sequences were aligned with other available *Paratylenchus* and a few outgroup sequences from GenBank using MAFFT [12]. The phylogenies were reconstructed in RAxML [13] implemented on the CIPRES Science Gateway [14].

### Genome assembly, annotation and functional prediction

The raw reads were quality checked using FASTP [15], and reads < 50 bp, with a Q-score < 20, or containing > 3 ambiguous bases (n), were discarded. De novo assembly was performed in Megahit [11]. The assembled genome was submitted to GenBank, with BioProject id: PRJNA1200215. The BlobToolKit 2.6.5 [16] was used to evaluate the quality of genome assembly and examine possible contamination. The assembly statistics were summarized in QUAST [17]. The completeness of the assembled genome was evaluated by BUSCO [18]. The genome size was estimated by k-mer counting implemented in Jellyfish [19] and GenomeScope 2.0 [20]. tRNA was predicted by tRNAscan-SE2 [21], and rRNA was predicted by barrnap (https://github.com/tseemann/barrnap). Gene prediction was performed in Braker3 pipeline [22]. Functions of these genes were annotated by eggNOG-mapper according to GO, KOG and KEGG databases [23]. The secreted proteins were predicated by SignalP 4.0 [24], and the selected genes were summarized to GO category in eggNOG-mapper. Since carbohydrate-active enzymes were closely related to nematode parasitism, the candidate genes were further annotated in dbCAN3 [25].

## Results

### Morphological and molecular species identification

**Fig. 1:**
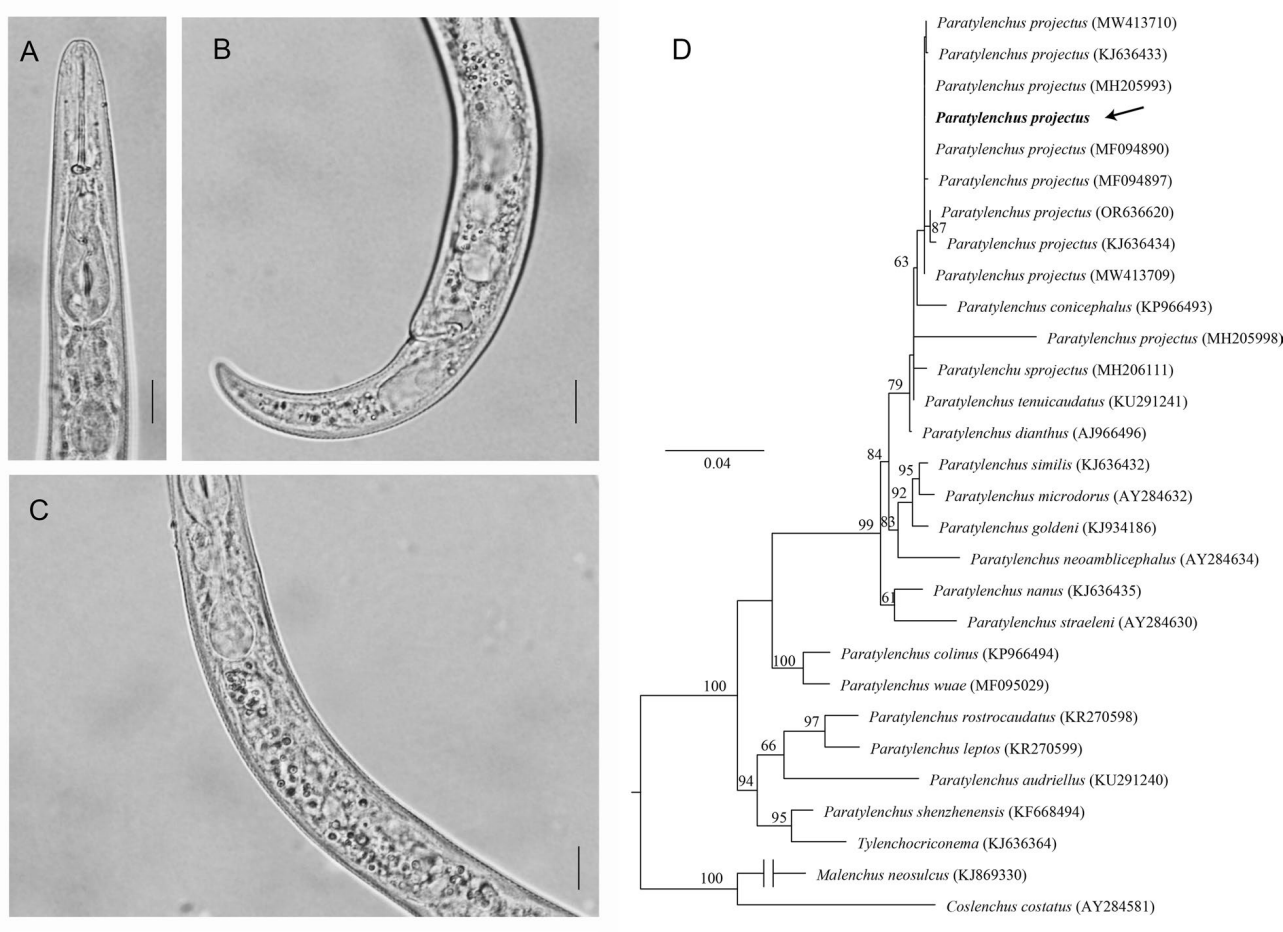
Morphological characteristics and phylogenic tree of *Paratylenchus projectus*. **A** Female anterior body region; **B** female tail region; **C** pharynx intestinal junction. Scale bar = 10 μm. **D** Maximum likelihood tree of *Paratylenchus projectus* based on 18S rRNA gene. The values at clade nodes indicate bootstrap; only those > 60 were given in nodes

The female body is slender and ventrally curved when heat-relaxed (Fig. 1A–C), with cuticle annulations not visible. The lateral field displays four distinct lines. The lip region is conoid and rounded, with a flattened anterior end, and the labial framework shows weak sclerotization. The stylet is straight with rounded knobs. The median pharyngeal bulb is slender and elongated, containing large sclerotized valves, while the basal bulb is pyriform. The excretory pore is located at or slightly anterior to the basal pharyngeal bulb. The hemizonid is 1–2 annuli long and positioned just anterior to the excretory pore. Both the spermatheca and crustaformeria are well developed, with the spermatheca being rounded. The vulva appears as a transverse slit occupying half the body width, with prominent lips; the anterior lip protrudes further than the posterior, and vulval flaps are present. The tail is slender, conoid and finely annulated, and it gradually tapers to form a subacute terminus.

The 18S rRNA showed 99.76% similarity to *P. projectus* (MF094890) with four differences in nucleotides. The phylogeny (Fig. 1D) placed the new sequence together with other *P. projectus* sequences in the same clade as sister to *Paratylenchus conicephalus* (KP966493). Likewise, the 28S rRNA sequence is 99.06% similar to *P. projectus* (MW413656) with seven differences in nucleotides. The phylogeny placed the new sequence within a clade including three closely related species: *P. projectus*, *Paratylenchus nanus* and *Paratylenchus neoamblicephalus* (Fig. S1). In ITS, the most similar sequence was *P. projectus* (OQ749699), with 99.05% similarity (six difference in nucleotides) and placed as a sister to the new sequence (Fig. S2)*.*

### Genome sequencing and gene annotation

**Fig. 2:**
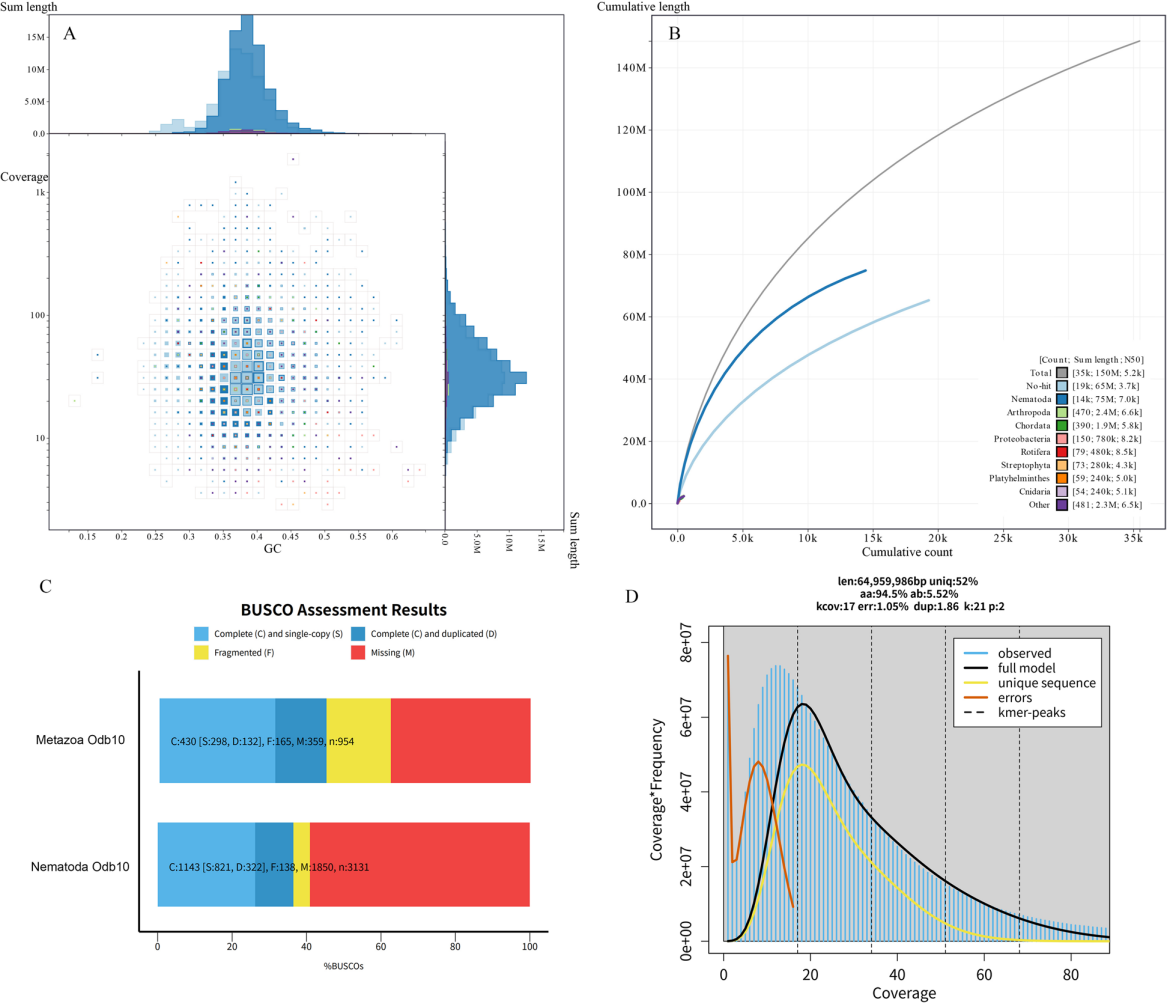
Genome assembly statistics. **A** Phylum-level-annotated GC-coverage plots; **B** cumulative length for assembly of *Paratylenchus projectus.* **C** BUSCO estimation of genome completeness using Metazoa Odb10 and Nematoda Odb10 databases; **D** genome size estimated by k-mer counting

A total of 47-Mb raw reads were obtained with Illumina sequencing. After quality control, 45-Mb clean reads were used for genome assembly. The assembly statistics are shown in Table 1. In general, the assembly was largely fragmented. There were 84,313 contigs for a total of 191.36-Mb genome length, with the longest contig being 68.99 Kb, and 167.7 Mb left after removing short contigs (< 1000 bp). The BUSCO analysis suggested 45.1% and 36.5% completeness using Metazoa Odb10 and Nematoda Odb10 databases (Fig. 2C). A further kmer-based statistical approach estimated genome size to be 64.9 Mb, significantly smaller than the assembled size (Fig. 2D).

**Table 1:** Genome assembly statistics of *Paratylenchus projectus*

Characterization	Data
Total length(Mb)	191.36
Number of contigs	84,313
Contigs (≥ 10,000 bp)	2348
Largest contig (bp)	68,986
N50 (bp)	3685
N90 (bp)	898
GC (%)	38.24

To further examine possible contamination, the filtered contigs were plotted by sequencing depth and GC content (Fig. 2A) and a cumulative sequence length (Fig. 2B). In general, contamination was limited. Among 150 Mb were annotated contigs, and 50% (75 Mb) were assigned to Nematoda. Although a total of 65 Mb contigs were not identified, they were probably nematodes, as nematode reference sequences were scarce in the database. Only a small portion of data were identified as contamination, e.g. Arthropoda. The distribution of sequences in the GC-Coverage quadrant indicates that the GC content of the genome data for the new species *P. projectus* ranges between 35 and 40%, with a coverage of around 55 ×. Most sequences that failed to align with the database or were annotated as “Other” also fall within this range, suggesting that the lack of nematode genome information in the database might have led to nematode sequences being unannotated or misannotated as “Other.” Figure 2B indicates that the sequences annotated as Nematoda are the longest, totaling 14,000 sequences with an N50 value of 7.0 Kb. The sequences annotated as “No-hit” are shorter, totaling 19,000 sequences. This suggests that at least 90% of the nematode sequences are present in the genome, indicating good assembly quality.

**Fig. 3:**
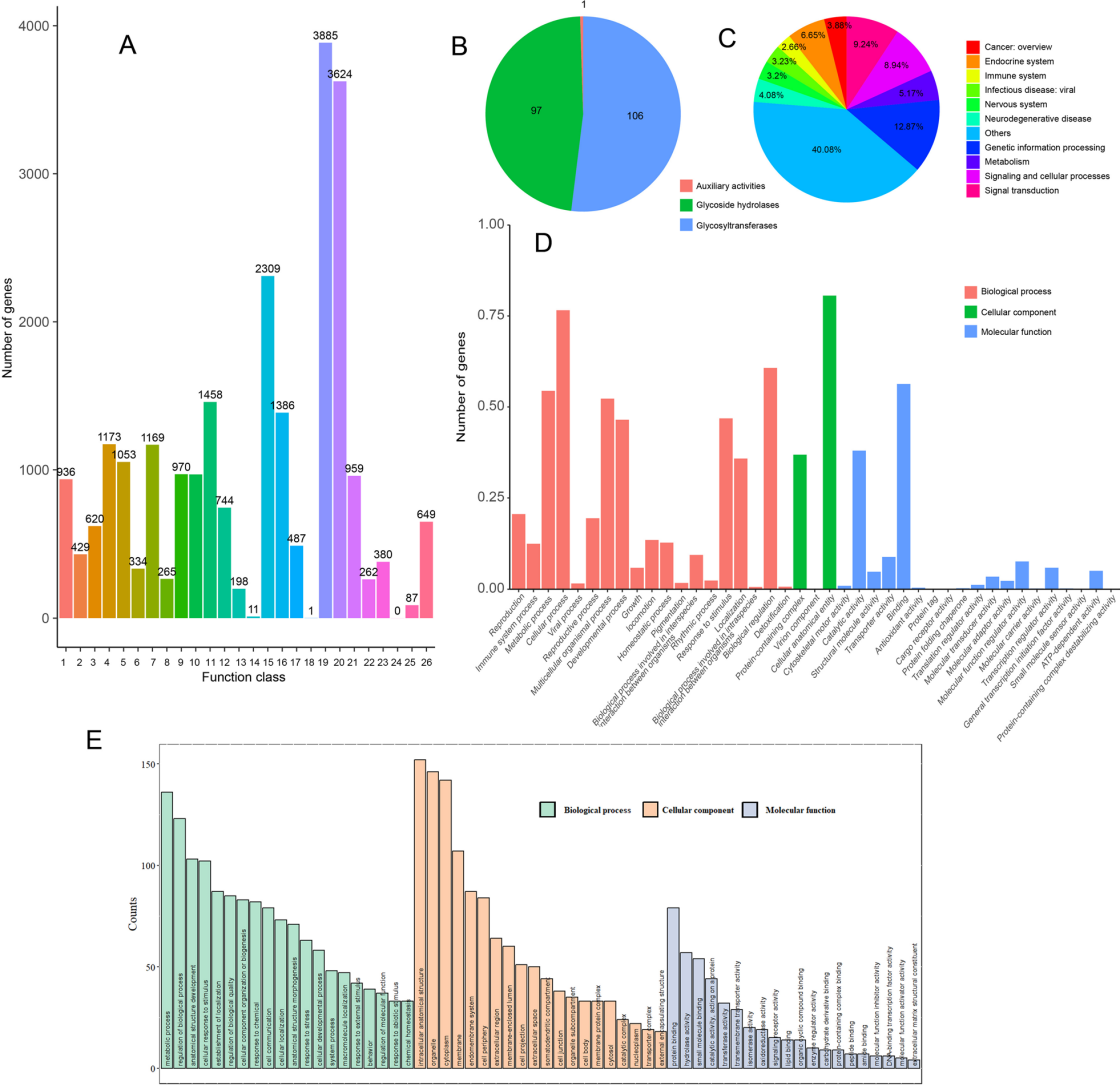
Gene function annotation of *Paratylenchus projectus*. **A** COG annotation; **B** Carbohydrate-active enzyme annotation; **C** KEGG annotation; **D** GO annotation; **E** the estimated signal peptides assigned to different functional groups by GO. The numbers on the x-axis in COG annotation represent: (1) RNA processing and modification; (2) chromatin structure and dynamics; (3) energy production and conversion; (4) Cell cycle control, cell division, chromosome partitioning; (5) amino acid transport and metabolism; (6) nucleotide transport and metabolism; (7) carbohydrate transport and metabolism, (8) coenzyme transport and metabolism; (9) lipid transport and metabolism; (10) translation, ribosomal structure and biogenesis; (11) transcription; (12) replication, recombination and repair; (13) cell wall/membrane/envelope biogenesis; (14) cell motility; (15) posttranslational modification, protein turnover, chaperones; (16) inorganic ion transport and metabolism; (17) secondary metabolite biosynthesis, transport and catabolism; (18) general function prediction only; (19) function unknown; (20) signal transduction mechanisms; (21) intracellular trafficking, secretion and vesicular transport; (22) defense mechanisms; (23) extracellular structures; (24) mobilome: prophages, transposons; (25) nuclear structure, (26) cytoskeleton

The predicted genes were annotated using GO, COG, KEGG and dbCAN3 databases (Fig. 3). For dbCAN3, among the annotated genes, the most abundant were glycosyltransferases (GT), with 106 related genes, followed by glycoside hydrolases (GH) with 97 related genes, and auxiliary activities (AA) only have one annotated gene (Fig. 3A). The KEGG (Fig. 3B) revealed that 12.87% of the genes were associated with genetic information processing, followed by 9.24% related to signal transduction-related genes and 8.94% linked to signaling and cellular processes. A total of 3885 genes were annotated as having unknown functions in the COG analysis (Fig. 3C), suggesting that this nematode is distantly related to other sequenced species. Among the remaining genes, those involved in signal transduction mechanisms were the most abundant, with 3624 genes identified, followed by 2309 genes related to posttranslational modification, protein turnover and chaperones.

In GO annotation (Fig. 3D), the most abundant biological processes (BP) were cellular process (GO:0009987), biological processes involved in intraspecies interaction between organisms (GO:0051703) and metabolic processes (GO:0008152) involving 76.58%, 60.73% and 54.42% of the genes, respectively. For cellular components (CC), the most represented categories were cellular anatomical entity (GO:0110165), protein-containing complex (GO:0032991) and virion component (GO:0044423), involving 80.60%, 36.88% and 0.03% of the genes, respectively. Regarding molecular functions (MF), the most abundant were binding (GO:0005488), catalytic activity (GO:0003824) and transporter activity (GO:0005215), encompassing 56.32%, 38.00% and 8.79% of the genes, respectively. Among annotated genes, 3184 were identified as signal peptides that had potentially been secreted by nematodes, and these genes were assigned to different categories (Fig. 3E).

**Fig. 4:**
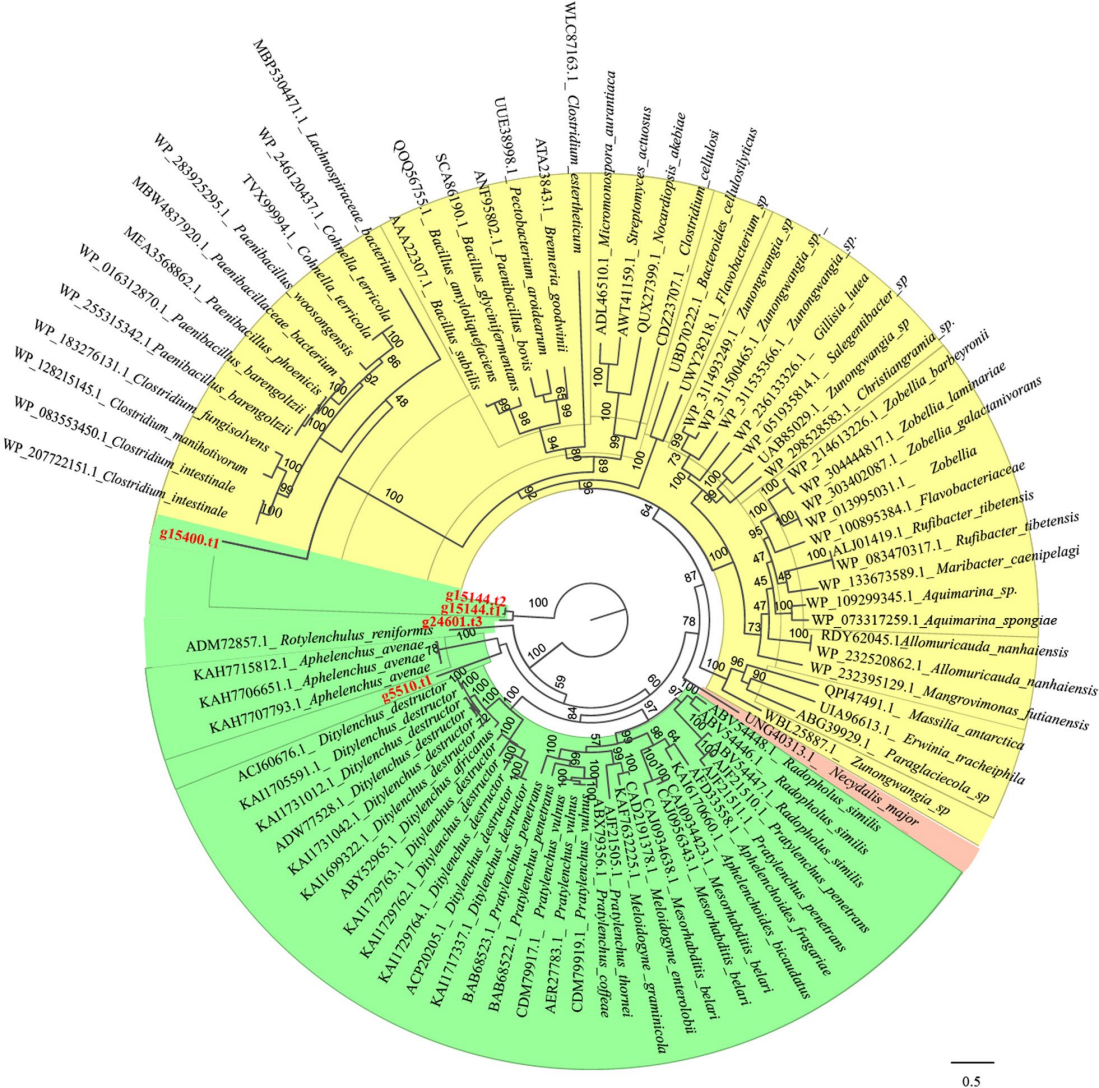
Maximum likelihood phylogeny of predicted GH5 family using protein sequences. The bootstrap values are indicated in nodes. The colors modules in the tree show the taxonomy of sequences. Yellow represents bacteria, green represents nematodes, and pink represents insects. The sequences from *Paratylenchus projectus* are indicated in red. The scale bar indicates expected changes per site

Genes belonging to the GH5 family were extracted from the *P. projectus* genome. The recovered genes were grouped into three distinct clades (Fig. 4). The first clade, which was fully supported (posterior probability [PP] = 1), contained two genes: G24601.t3 and G15144, with G15144.t1 and G15144.t2 representing two alternative splicing variants of the same gene. This cluster was positioned as basal in the GH5 phylogeny, showing close relation to other nematodes. The gene G5510.t1 formed the second clade, appearing as a sister group to the fungivorous nematode *Aphelenchus avenae*. The third clade contained only one gene, G15400.t1, which was a sister to a moderately supported clade (PP = 0.48) that included three fungal genera—*Cohnella*, *Paenibacillus* and *Clostridium*—as well as two species from the unidentified families Lachnospiraceae and Paenibacillaceae.

**Fig. 5:**
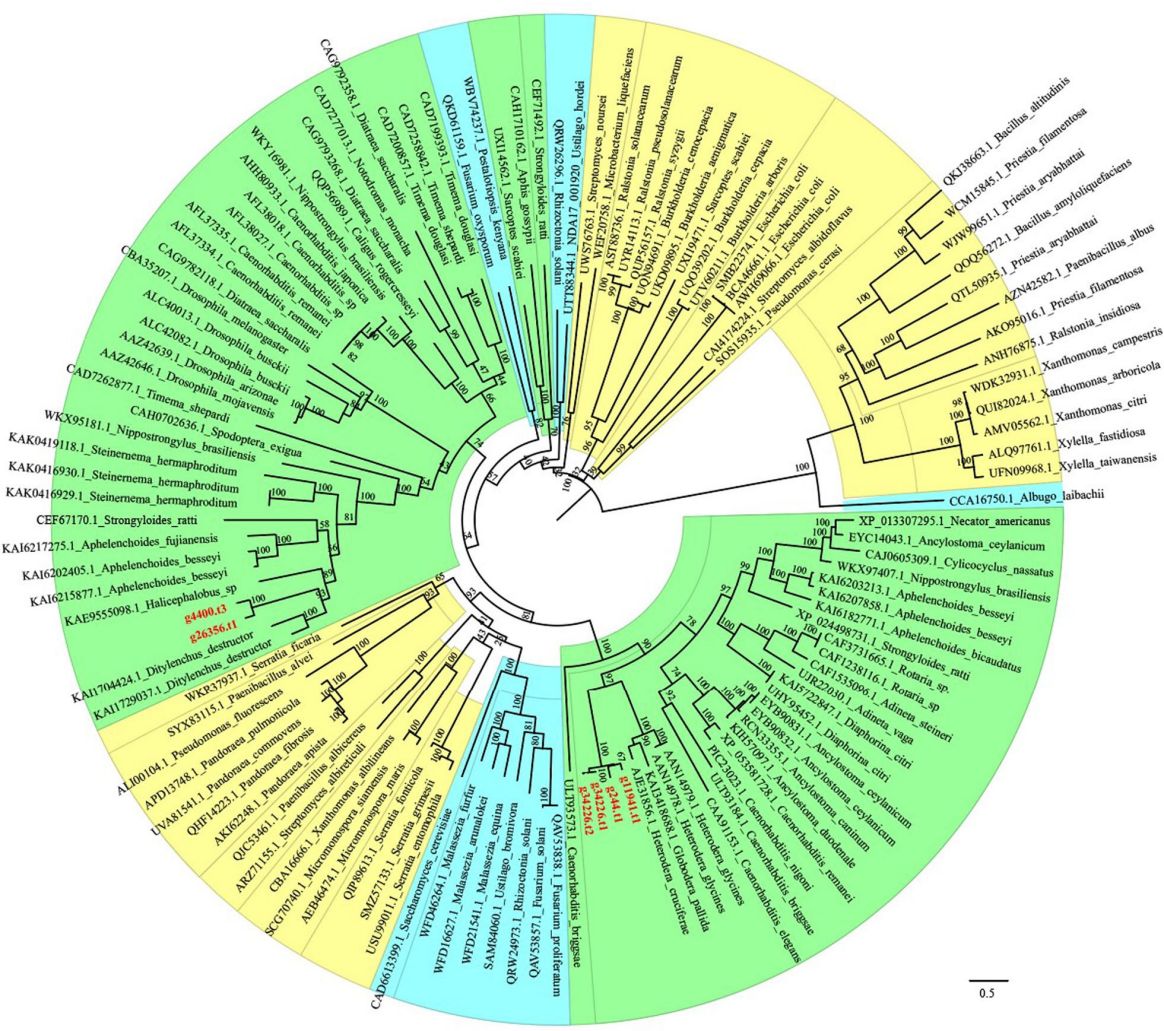
Maximum likelihood phylogeny of predicted GH18 family using protein sequences. The bootstrap values are indicated in nodes. The color modules in the tree show the taxonomy of sequences. Yellow represents bacteria, green represents nematode + protozoa + arthropods, and blue represents fungi. The sequences from *Paratylenchus projectus* are indicated in red. The scale bar indicates expected changes per site

Similarly, the GH18 chitinase genes in *P. projectus* were divided into two clades (Fig. 5). The first clade comprised four sequences: G11941.t1, G244.t1, G34226.t1 and G34226.t2, forming a fully supported clade (PP = 1) that was closely related to sedentary plant-parasitic nematodes of the genera *Heterodera* and *Globodera*. The second clade consisted of two sequences, G4400.t3 and G26356.t1, which were nested within another nematode clade and closely related to both the fungivorous *Ditylenchus destructor* and the bacterivorous *Halicephalobus*.

## Discussion

*Paratylenchus projectus* Jenkins, 1956, was initially described from root and soil samples in a pasture in Maryland [26] and was later found in highbush blueberries in Arkansas [27]. In this study, *P. projectus* was isolated from the rhizosphere of blueberry plants, marking the first report of this species associated with blueberries in China. The species identity was confirmed through morphological analysis and molecular phylogeny based on 18S, 28S and ITS rRNA sequences. Since blueberries thrive in acidic soils with a pH range of 4.0 to 5.5 [28, 29], this discovery suggests that *P. projectus* can tolerate acidic environments.

Of approximately 1 million estimated nematode species [30] and 28,537 described species [31], only a small fraction—275 species as of October 2024 (NCBI)—have been sequenced for their genomes. Moreover, sequencing efforts have been biased towards the model organism *Caenorhabditis elegans* and vertebrate parasites, leaving no genomic data available for *Paratylenchus* or members of the family Tylenchulidae. In this study, the genome of *P. projectus* was sequenced using the Illumina platform, resulting in 84,313 contigs with a total assembly size of 191.36 Mb. Although highly fragmented, the genome size is within the range reported for other nematode genomes, which vary from 42.0 to 720.0 Mb [32]. The *P. projectus* genome is comparable to the bacterivorous *Pristionchus pacificus* at 172.5 Mb [33] but larger than those of the pinewood nematode *Bursaphelenchus xylophilus* at 74.6 Mb [34], the potato cyst nematode *Globodera pallida* at 124.7 Mb [35] and the rice white-tip nematode *Aphelenchoides besseyi* at 50.3 Mb [36]. However, it is smaller than the root-knot nematode *Meloidogyne arenaria*, which has a 284.05-Mb genome [37].

Plant-parasitic nematodes are known to possess a suite of genes encoding plant cell wall-degrading enzymes. Such enzymes have been identified and/or characterized in several species, including those from the genera *Meloidogyne* [38], *Globodera* [39], *Heterodera* [40], *Radopholus* [41], *Bursaphelenchus* [42], *Rotylenchulus* [43] and *Aphelenchus* [44] as well as *Aphelenchoides* [44, 45]. In this study, we examined genes belonging to the GH5 and GH18 families. Within the GH5 phylogeny, g5510.t1 was grouped within the *Aphelenchus* clade but was distinct from other tylench species, which were more closely related to *P. projectus*. This pattern may be due to the presence of multiple GH5 copies in *P. projectus*, as has been observed in other parasitic nematode genomes [35]. Given the highly fragmented nature of our assembled genome, it is possible that additional copies more closely related to tylenchs are present but were not recovered. Alternatively, different GH5 genes may have existed in a common ancestor of aphelenchs and tylenchs, with subsequent differential loss occurring in *Aphelenchus*. Two other GH5 representatives in *P. projectus* either represent possible contamination due to their close relationship with fungi (15400.t1) or belong to a basal clade, potentially indicating another undescribed nematode GH5 cellulase (G15144.t1, G15144.t2, and G24601.t3).

GH18 chitinases catalyze the biodegradation of β-1,4 glycosidic bonds in amino polysaccharides via a substrate-assisted retention mechanism. This gene family is widely distributed across diverse species, with functions including tissue degradation and remodeling, nutrient uptake, invasion and pathogenesis, and immune response regulation [46]. It is not surprising that three GH18 genes in *P. projectus* (G11941.t1, G244.t1, G34226) are closely related to *Heterodera* and *Globodera*, with G34226.t1 and G34226.t2 being two alternative splicing variants of the same gene. However, the other two genes (G4400.t3 and G26356.t1) represent the only instance of an obligate plant-parasitic member in this nematode clade. Since other members of this clade are either bacterivorous or facultative plant parasites that can also feed on fungi, our findings suggest that the GH18 genes in this clade may have undergone secondary loss in sedentary endoparasitic nematodes. More complete nematode genomes, particularly from basal Tylenchomorpha, will help further delineate the relationships between these two evolutionary lineages of nematode GH18 chitinases.

## Conclusions

Based on morphological analysis and molecular barcoding, we identified *Paratylenchus projectus* from the rhizosphere of blueberry. Using the Illumina platform, the draft genome of *P. projectus* was sequenced and assembled, representing the first genomic data for both the genus *Paratylenchus* and the family Tylenchulidae to our knowledge.

## Supplementary Information

Supplementary material 1: Fig. S1. Maximum likelihood tree of *Paratylenchus projectus* based on 28S rRNA gene. The values at clade nodes indicate bootstrap; only those > 60 are given in the node. The newly obtained sequence is indicated in bold. The scale bar indicates expected changes per site.

Supplementary material 2: Fig. S2. Maximum likelihood tree of *Paratylenchus projectus* based on ITS rRNA gene. The values at clade nodes indicate bootstrap; only those > 60 are given in the node. The newly obtained sequence is indicated in bold. The scale bar indicates expected changes per site.

Supplementary material 3. The sequences of signal peptides in *Paratylenchus projectus.*

## Data Availability

No datasets were generated or analysed during the current study.
