# Supplementary figures and images for: Draft genome of pin nematode Paratylenchus projectus recovered from rhizosphere of blueberry
Source: Parasit Vectors. 2025 Feb 26;18:77. doi: 10.1186/s13071-025-06680-8 (PMC11863913; doi:10.1186/s13071-025-06680-8)

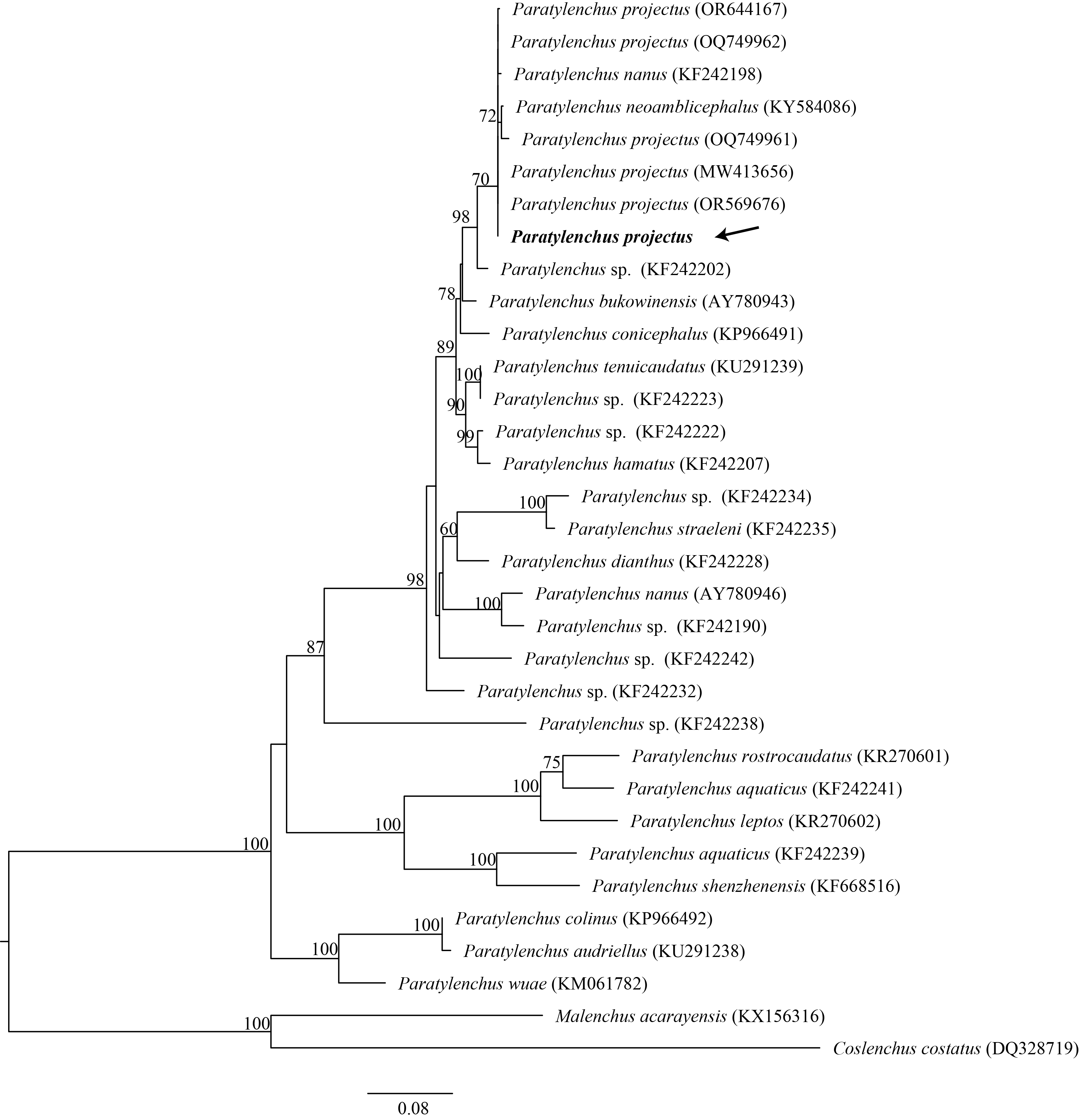

Supplement: Supplementary file 1 — Supplementary material 1: Fig. S1. Maximum likelihood tree of Paratylenchus projectus based on 28S rRNA gene. The values at clade nodes indicate bootstrap; only those > 60 are given in the node. The newly obtained sequence is indicated in bold. The scale bar indicates expected changes per site. [file 13071_2025_6680_MOESM1_ESM.jpg]

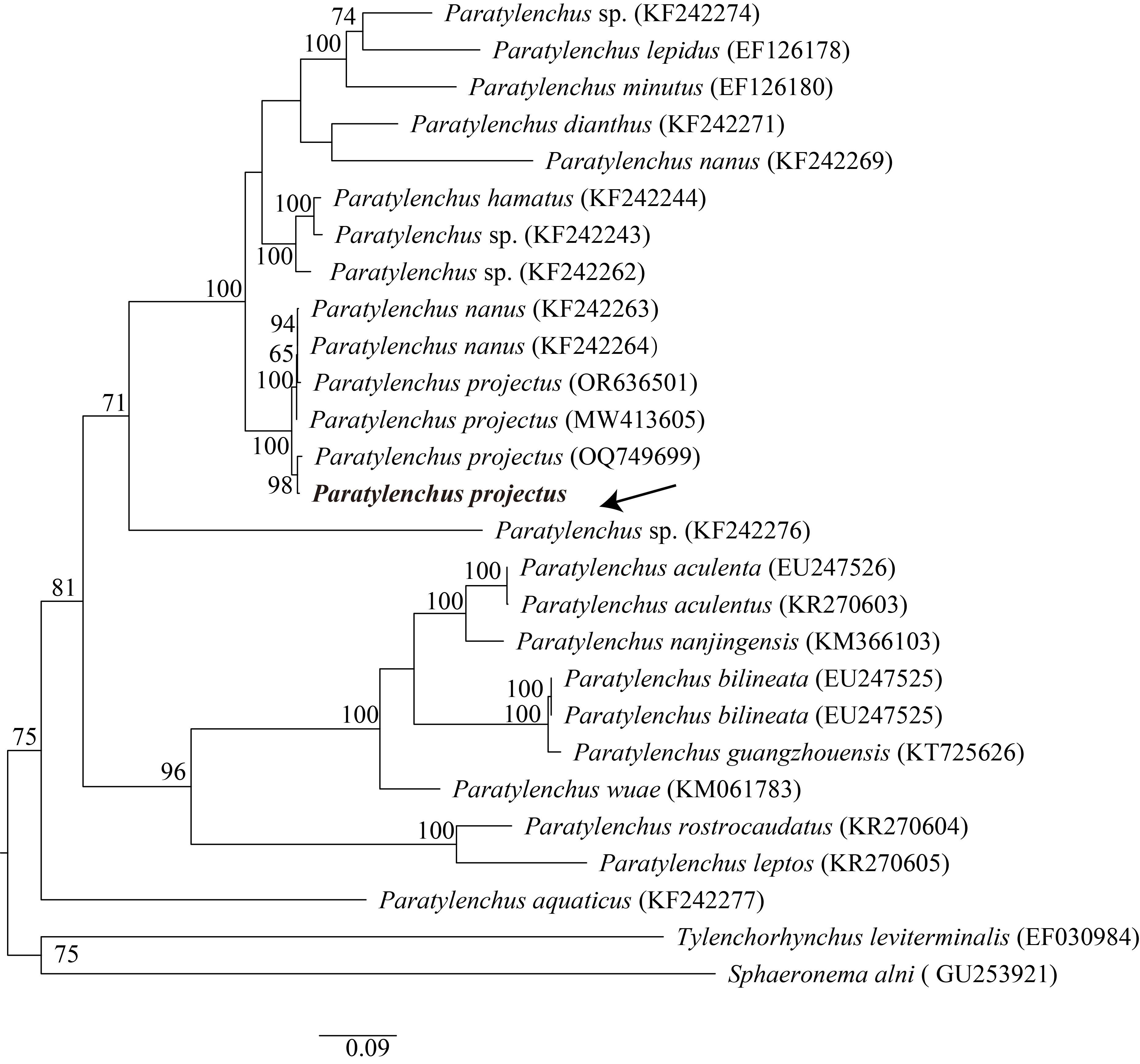

Supplement: Supplementary file 2 — Supplementary material 2: Fig. S2. Maximum likelihood tree of Paratylenchus projectus based on ITS rRNA gene. The values at clade nodes indicate bootstrap; only those > 60 are given in the node. The newly obtained sequence is indicated in bold. The scale bar indicates expected changes per site. [file 13071_2025_6680_MOESM2_ESM.jpg]
